# Compositional and Micro-Morphological Characterisation of Red Colourants in Archaeological Textiles from Pharaonic Egypt

**DOI:** 10.3390/molecules24203761

**Published:** 2019-10-18

**Authors:** Diego Tamburini, Joanne Dyer, Patrizia Davit, Maurizio Aceto, Valentina Turina, Matilde Borla, Marie Vandenbeusch, Monica Gulmini

**Affiliations:** 1 Department of Scientific Research, The British Museum, Great Russell Street, London WC1B 3DG, UK; DTamburini@britishmuseum.org (D.T.); JDyer@britishmuseum.org (J.D.); 2 Dipartimento di Chimica, Università degli Studi di Torino, Via Giuria, 7-10125 Torino, Italy; patrizia.davit@unito.it; 3 Dipartimento di Scienze e Innovazione Tecnologica, Università degli Studi del Piemonte Orientale, viale T. Michel, 11-15121 Alessandria, Italy; maurizio.aceto@uniupo.it; 4 Fondazione Museo delle Antichità Egizie, Via Accademia delle Scienze, 6–10123 Torino, Italy; valentina.turina@museoegizio.it; 5 Soprintendenza Archeologia belle arti e paesaggio per la città metropolitana di Torino, Piazza S. Giovanni, 2-10122 Torino, Italy; matilde.borla@beniculturali.it; 6 Department of Egypt and Sudan, The British Museum, Great Russell Street, London WC1B 3DG, UK; mvandenbeusch@britishmuseum.org

**Keywords:** natural dyes, Pharaonic Egypt, archaeological textiles, colour fastness, multi-analytical approach, mordants

## Abstract

When the imagination conjures up an image of an Egyptian mummy, it is normally one of a human body wrapped with undyed linen bandages. However, the reality was much more colourful, as shown by the set of red mummy shrouds and textile fragments from Pharaonic Egypt considered in this work. The textiles were subjected to scientific investigation with the main aim of shedding light on the sources of red colour and on the possible reasons for the different levels of colour fading. The red colourants were investigated using various non-invasive and micro-invasive approaches. The results pointed towards the presence of three sources of red colour, which, in increasing order of lightfastness, are safflower (*Carthamus tinctorius*), madder (*Rubia* spp.), and red ochre. Micro-morphological observations and elemental analyses also enabled some hypotheses to be formulated regarding the application of these colourants to the textiles. The results not only deepen our knowledge of dyeing technologies in ancient Egypt and shed new light on the function of red shrouds and textiles as part of the funerary practices of Pharaonic Egypt, but are also essential in planning the display and future preservation of these mummies and their associated textiles.

## 1. Introduction

The image that we commonly have of an Egyptian mummy is one of a human body wrapped with pallid-coloured linen bandages, made from natural flax fibres. While most bandages were made of undyed pieces of cloth, sometimes showing evidence of reuse, the external wrappings were regularly covered by a shroud. This usually consisted of a single length of cloth and was sometimes inscribed, dyed and/or painted [1].

**Figure 1:**
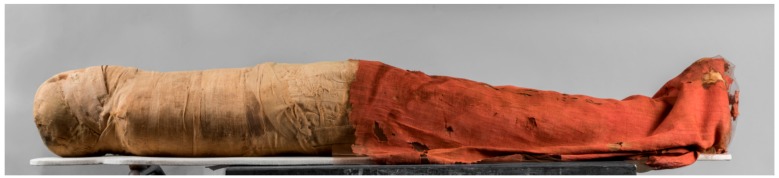
The red shroud partially covering the mummy of Nesamonendjam (registration number: Suppl. 5227/2; photo: Museo Egizio, Torino, Italy).

In particular, some mummies were covered by red shrouds, which in some instances have fully retained their intense colour over time (Figure 1). In other cases, damage, or the incidental removal of other bindings on the mummy, have exposed red-coloured textiles under a layer of faded ones, suggesting the original colourful appearance of the wrappings.

Textiles were of high importance in funerary practices and were often part of the goods chosen to accompany the dead in the afterlife [2]. In the Pharaonic period, flax was the most commonly used fibre for textile production. The majority of the garments represented in Egyptian paintings are white, in keeping with the fact that many of the surviving textiles from the Pharaonic period are undyed. Similarly, hundreds of mummies coming from Egypt are wrapped in multiple layers of undyed linen bandages. Nevertheless, the use of coloured threads, bands, and stripes decorated with blue or red threads, in combination with natural undyed ones, has a long tradition [1,3]. Entirely red textiles, although rare, are known from the Old Kingdom, and the use of a red shroud became a more frequent burial custom from the New Kingdom [4,5,6,7]. A piece of “dark red linen from Sais” is mentioned in the Ritual of Embalming, involving the wrapping of fingers and toes of the mummy [8,9,10]. In the context of ancient Egypt, the colour red can have different and ambivalent meanings. It was often believed to have an association with evil beings and was, for example, used to attract the reader’s attention toward a potentially dangerous hieroglyph in written texts. It could symbolise life and regeneration and could be associated with diverse deities such as Isis, Sekhmet, and Hathor. It is also related to Ra and the sun. Echoing the daily rise of the solar disc, red encompassed the notion of regeneration [11]. The god Osiris is frequently depicted in a red shroud juxtaposed with bead-net patterns. Red could also represent anger, chaos, and fire and, in this case, it is closely associated with Seth [11,12]. The practice of covering the body with a red shroud could also be related to the use of the colour red as a background on coffins and for the contiguous edges of the lid and the coffin case [13]. The purpose appears to be both apotropaic and restorative: the mummy was wrapped in a red shroud that could be covered with a bead-net in order to protect the body and facilitate the rebirth of the deceased in the afterlife, following Osiris’ path.

The chemical analysis of richly coloured Egyptian textiles from the first millennium CE has a long-standing tradition [14,15,16,17], whereas less attention has been given to the systematic investigation of the dyes used in Pharaonic textiles from the first millennium BCE with state-of-the-art scientific techniques. The published studies [1,4,18,19,20] indicate that sources of red colourants used to dye textiles were mainly ochreous earths and plant dyes. Madder (*Rubia* spp.) was probably the most important dye of plant origin in Pharaonic Egypt, and safflower (*Carthamus tinctorius*), henna (*Lawsonia alba* or *Lawsonia inermis*), alkanet (*Anchusa tinctoria*), and saffron (*Crocus sativus*) are also mentioned as possible sources of dyes.

Scientists can presently rely on a series of techniques for investigating the colourants in archaeological textiles, each exhibiting specific potential and/or limitations for the recognition of the dyes and for the interpretation of dyeing technologies. Normally, the in-depth investigation of textiles in museum and historic collections adopts protocols involving a set of chemical and physical techniques. The investigation generally starts with a non-invasive inspection of some selected spots by means of fibre optic reflectance spectroscopy (FORS) and/or portable fluorimetry (p-FL) [17,21,22,23]. However, research in the field is rapidly developing and, recently, the merits of multispectral imaging for obtaining a holistic insight into the chemical nature of the materials composing archaeological textiles have been discussed [16,23]. These approaches allow preliminary information on the colourants to be obtained and can guide subsequent micro-sampling to further deepen the level of the information on the colouring materials present. Samples could then be investigated by surface enhanced Raman spectroscopy (SERS), which requires a very small sample (a few particles) and may produce information at the molecular level [24,25,26]. Presently, the most effective and selective characterisation of the organic colourants in the yarns is obtained by high-performance liquid chromatography mass spectrometry (HPLC-MS), which may lead to the identification of the colourant sources as being from plants or insects, often to species level [23,27,28,29,30], provided that a micro-sample (2–3 mm of a thread) from the textile can be sacrificed.

In addition to the analysis of the organic dyestuffs, the characterisation of the inorganic composition of the yarns gives information on the metallic mordants that were employed to fix the colour and reveals further details on ancient dyeing technologies. Elemental analytical techniques, namely energy dispersive X-ray spectrometry coupled to scanning electron microscopy (SEM-EDS), particle-induced X-ray emission (PIXE), and laser-induced breakdown spectroscopy (LIBS) have been employed to characterise the mordanting ions [31,32,33,34].

In this work, a systematic, multi-technique investigation was used to shed light on the dyeing materials and techniques employed for colouring red textiles in Pharaonic Egypt. The investigation focused on mummies with red shrouds and wrappings—including partially faded examples—and fragments of red textiles selected from the collections at the Museo Egizio (Torino, Italy) and at the British Museum (London, UK), dated from the New Kingdom (the oldest one is S.1698 from Deir el-Bahry cachette) to the Late Period of ancient Egypt (ca. 1069-525 BCE). The mummies and textiles considered are briefly described in Table 1. The labels used henceforth to indicate the samples—and their correspondence with the archaeological textiles—are also reported in Table 1.

The archaeological textiles were investigated non-invasively by FORS and p-FL. Micro-samples were then taken and imaged by optical microscopy (OM) under visible and UV illumination. The samples were also analysed by micro-XRF, SEM-EDS, and finally HPLC-MS. The analytical approach adopted has been primarily aimed at highlighting the advantages and drawbacks of each technique regarding the identification of colourants and dyeing methods used in red Pharaonic textiles. The investigation of inorganic mordants was also considered, particularly as these textiles are normally heavily contaminated and elemental analyses can produce data that may at times be misleading. 

Overall, the main aim of the study was to establish an analytical baseline over which further case studies may be assessed and added to in future. This will provide archaeologists with important information to assess how the use of red shrouds and textiles was embedded in the funerary practices of ancient Egypt. In addition, these data will allow museum curators to more knowledgably plan the display and preservation of this precious, fragile, and often light-sensitive archaeological evidence.

## 2. Results

Table 2 summarises the results obtained by each technique and indicates which analyses have been performed for each of the considered archaeological textiles (or samples).

### 2.1. Identification of the Main Colourants

#### 2.1.1. FORS and p-FL

In order to ease the direct comparison with the results obtained with the other (invasive) techniques, the labels of the samples taken from the textiles (see Table 1) are used in the discussion of these results. However, these analyses were performed directly on the textiles without the need for invasive sampling.

For a large majority of the textiles investigated by FORS, the spectroscopic features recorded were of suitable quality to reliably identify the main colourants, provided that the analytical spot targeted areas where the colour was still appreciable with the naked eye.

**Figure 2:**
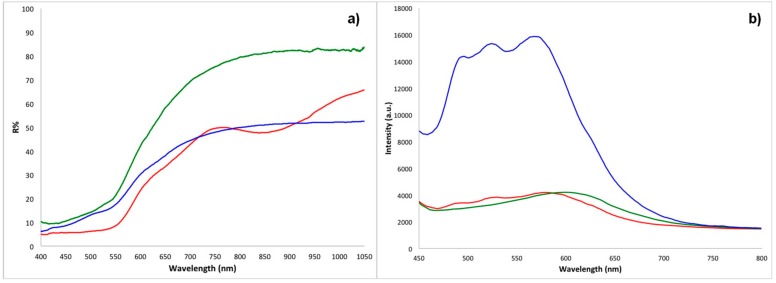
(**a**) Representative fibre optic reflectance spectroscopy (FORS) spectra (R = reflectance) and (**b**) representative portable fluorimetry (p-FL) spectra for textiles dyed with ochre (red), madder (green), and safflower (blue). FORS spectra: ME7, BM4, and ME3. p-FL spectra: ME7, ME2, and ME5.

For heavily faded regions of the textiles, for example samples ME4 and ME6, only the spectral features of undyed flax fibres were detected. For all the other measurements, diffuse reflectance spectra highlighted three different scenarios. In samples ME7 and BM1, the characteristic absorption features of red ochre were evident, with the two inflection points around 575–590 nm and 700 nm, the characteristic positive slope in the region above 600 nm, and the absorption band due to ligand field transition centred around 850–900 nm [36,37]. In BM2, BM4 and ME2, the absorption peaks of madder at 505–515 nm and 540–550 nm were detected [16,21,23]. In BM3, ME1, ME3 and ME5, an absorption band, centred at about 530 nm, was indicative of the presence of red safflower [23,37,38]. 

p-FL spectra performed on selected textiles (Table 2) further supported the identifications obtained by FORS. The textiles dyed with red safflower showed a characteristic intense emission band at ca. 570 nm [38], whereas madder produced a characteristic spectrum with an intense emission band at ca. 600 nm [21]. Fluorimetry was not conclusive for the identification of the red ochre. Representative FORS and p-FL spectra for the above-described scenarios are reported in Figure 2.

#### 2.1.2. HPLC-ESI-Q-ToF

The results obtained for the in-situ non-invasive approach were confirmed and, in some cases, complemented by high-performance liquid chromatography coupled to electrospray ionisation and quadrupole time-of-flight (HPLC-ESI-Q-ToF).

Three samples did not show any signals ascribable to dye molecules in their chromatographic profiles. In one case, namely sample ME4, the thread was totally faded, suggesting that any material trace of the original colour, which is still visible in other parts of the textile, had been lost. Additionally, FORS and p-FL spectra did not give any indication of the colourants once present in this faded region of the textile. The other two samples, namely ME7 and BM1, were instead intensely red-coloured. In these cases, the chromatographic approach was not adequate for the detection of these colourants because of the inorganic nature of the red compounds, which were instead easily detected and characterisable by the non-invasive approach.

**Figure 3:**
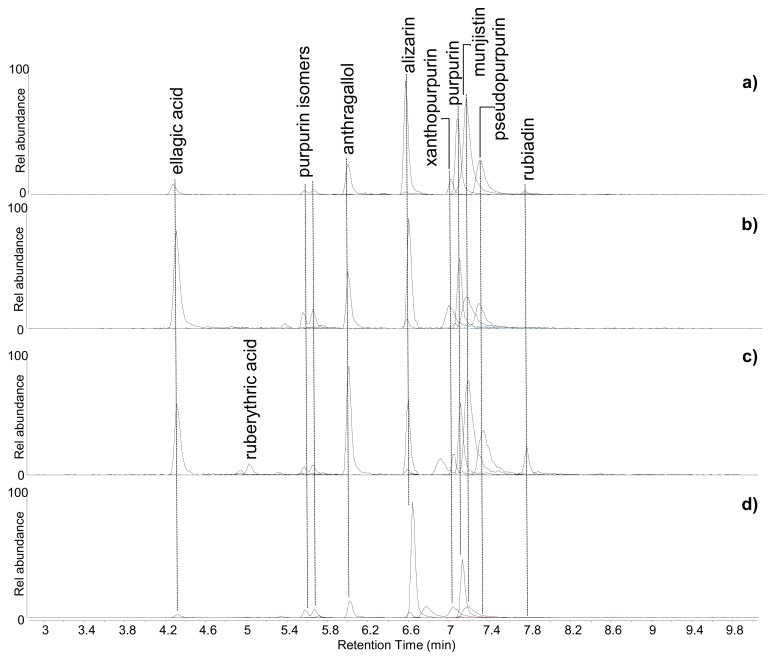
Extract ion chromatograms of the molecular components of madder (*Rubia* spp.)—alizarin and xanthopurpurin [M-H]^−^ = 239.035 *m/z*; rubiadin [M-H]^−^ = 253.051 *m/z*; purpurin and anthragallol [M-H]^−^ = 255.031 *m/z*; munjistin [M-H]^−^ = 283.025 *m/z*; pseudopurpurin [M-H]^−^ = 299.020 m/z; ruberythric acid [M-H]^−^ = 563.1406 *m/z*—and tannin-related compound—ellagic acid [M-H]^−^ = 300.999 *m/z*—obtained by HPLC-ESI-Q-ToF analysis (negative mode) of (**a**) ME2, (**b**) BM2, (**c**) BM4, and (**d**) BM5.

HPLC-MS data indicated that four samples, namely ME2, BM2, BM4, and BM5, were dyed using madder (*Rubia* spp.). Three out of these four samples (ME2, BM2, BM4) were included in the preliminary non-invasive survey, and madder was detected from the FORS and p-FL (only for ME2) spectra. The chromatographic profiles (Figure 3) highlighted the presence of several anthraquinones in these samples. Among them, alizarin, purpurin, xanthopurpurin, munjistin, pseudopurpurin, anthragallol, and two other isomers of purpurin were detected in all samples. Rubiadin was only detected in samples ME2 and BM4. The latter sample also contained ruberythric acid.

The high relative abundance of alizarin, generally found in all the samples, pointed towards the use of *Rubia tinctorum* as the possible source of madder [39,40]. However, the straightforward identification of the *Rubia* species used in archaeological samples is a challenging task, as the natural variability in the chemical composition of the madder root, ancient extraction and preparation procedures, ageing, and the analytical protocol adopted can significantly modify the original dyestuff composition and make the interpretation difficult [30,41]. In fact, even among these samples, the distribution and relative abundance of the madder components were quite variable, as shown in Figure 3.

Ellagic acid, related to the presence of tannins, was identified in all samples and was particularly abundant in samples BM2 and BM4. Red anthraquinones from madder have poor chemical affinity with textile fibres. The use of a mordant is generally necessary and both metallic ions and organic compounds (among them tannins) have been used to this end, therefore the tannin-related compound detected in the samples may point towards the use of tannins to favour the interaction between the coloured chemical species and the flax fibres, and perhaps also to modify the final red shade [42].

**Figure 4:**
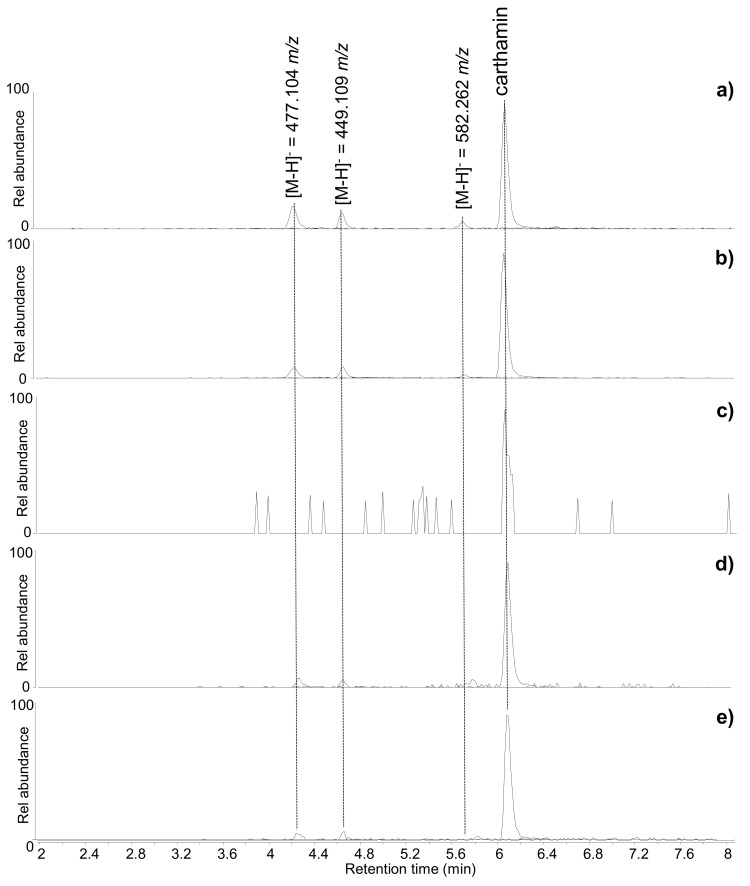
Extract ion chromatograms of the molecular components of safflower (*C. tinctorius*)—carthamin [M-H]^−^ = 909.210 *m/z*; carthamin degradation products [M-H]^−^ = 449.109 and 477.104 *m/z*; non-coloured safflower component [M-H]^−^ = 582.262 *m/z*—obtained by HPLC-ESI-Q-ToF analysis (negative mode) of (**a**) ME1, (**b**) ME5, (**c**) ME6 (the chromatographic peak for carthamin—detected in trace amounts—is only slightly above the level of instrumental noise, as observed from the surrounding background peaks), (**d**) BM3, and (**e**) BM6.

In six samples, namely ME1, ME3, ME5, ME6, BM3, and BM6, HPLC-MS analysis (Figure 4) showed the presence of safflower (*Carthamus tinctorius*).

Among these, five had been considered in the preliminary non-invasive spectroscopic analyses and the spectroscopic features of red safflower were detected in three of them. In the heavily faded ME6, the dye was not detectable by FORS and p-FL, but it was identified by the chromatographic approach, highlighting the higher sensitivity of HPLC-MS over FORS and p-FL. The identification of safflower by HPLC-MS was achieved through the detection of carthamin and/or its degradation products (Figure 4). Carthamin is the main molecular species responsible for the red colour of safflower, but, as it easily undergoes chemical and photo-degradation, it is usually found in the co-presence of two degradation products, which are the result of a reverse aldol condensation [39,43,44]. These are chalcone derivatives with chemical formulae C_21_H_22_O_11_ and C_22_H_22_O_12_, respectively, and the masses of their deprotonated ions [M-H]^−^ are 449.109 and 477.104 *m/z,* respectively. Another non-coloured component, whose [M-H]^−^ is 582.262 *m/z* (probably corresponding to the chemical formula C_21_H_45_NO_17_), is often reported in safflower samples [38,39,43]. These four molecules were detected in five out of the six samples containing safflower (Figure 4), with the very faded sample ME6 showing only traces of carthamin. All the other samples showed similar chromatographic profiles, with carthamin present as the most abundant peak and slightly variable relative abundances of the carthamin degradation products and the compound with [M-H]- = 582.262 *m/z*. The relatively high abundance of carthamin is indicative of a good state of preservation of the dye, which is in agreement with these samples being taken from areas protected from light and showing a relatively intense orange/pink colour.

### 2.2. Micro-Morphology and Elemental Composition

In addition to the spectroscopic and chromatographic approaches, the fibres were analysed by other methods, in order to highlight additional selective features for the tentative identification of the colourants and to formulate hypotheses on the dyeing methods employed.

#### 2.2.1. Optical Microscopy Under Visible and UV Light

Results from the spectroscopic and chromatographic approaches were employed to guide the interpretation of the images obtained by optical microscopy and enhanced our capability to retrieve the underlying information.

**Figure 5:**
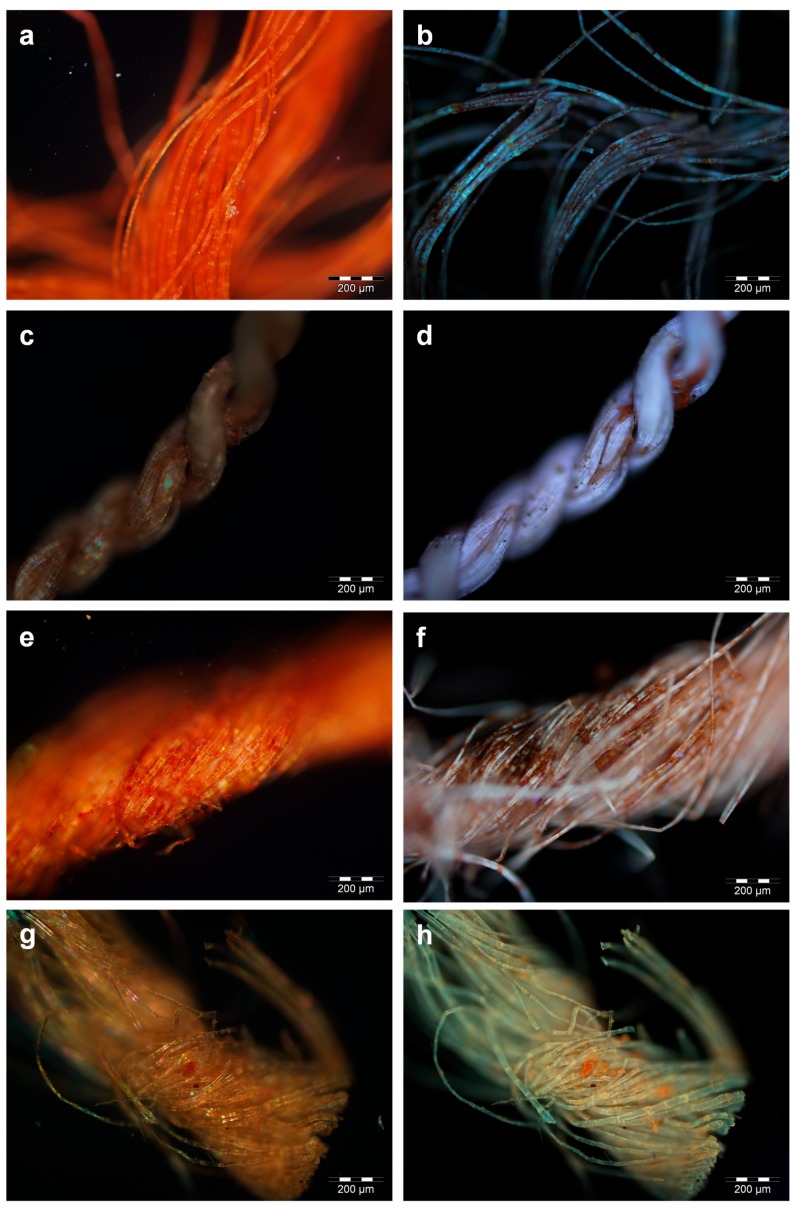
Optical microscopy (OM) images of sample ME7 (red ochre) as observed under visible (**a**) and UV (**b**) illumination; OM images of sample BM5 (madder and tannins) as observed under visible (**c**) and UV (**d**) illumination; OM images of sample ME2 (madder and tannins) as observed under visible (**e**) and UV (**f**) illumination; OM images of sample BM6 (safflower) as observed under visible (**g**) and UV (**h**) illumination.

For samples ME7 and BM1, in which red ochre was identified, the fibres appeared almost homogeneously red in vis-OM (Figure 5a), whereas a fine dispersion of absorbing particles was clearly observed under UV illumination (Figure 5b). This is in agreement with the UV-absorption properties of red ochre [45]. Lignocellulosic fibres, such as flax, are known to appear off-white/beige under UV illumination [16] and an intense off-white emission is indeed observed where the pigment particles are not present.

Flax fibres dyed with madder appeared red under visible illumination (Figure 5c), whereas coloured agglomerates up to 50 µm in length became evident under UV illumination, due to their characteristic orange luminescence (Figure 5d). Sample ME2 showed the highest abundance of these coloured agglomerates, which appeared brick-red under visible light (Figure 5e) and produced a dark orange hue under UV illumination (Figure 5f). As the light emitted under UV excitation resulted from the combined contribution of the emissive anthraquinones, present with variable relative abundances in the yarns (Figure 3), a slight variability in intensity and colour was expected. In particular, alizarin, which was generally detected with high relative abundance compared to the other anthraquinones, would contribute to the orange hue of the luminescence. The contribution of the UV-absorbing qualities of the tannins should also be considered. In fact, the other samples (not shown) exhibited slightly different luminescence intensities, although the orange hue of madder was distinguishable in all cases.

All the samples dyed with safflower exhibited a more limited number of smaller (10–20 µm) coloured particles with respect to the madder-containing ones, and a general pink/orange hue was observed throughout some of the fibres in the vis-OM images (Figure 5g). The particles and the fibres produced luminescence under UV illumination from bright to less intense orange (Figure 5h), following the extent of fading, as is typical of safflower [39].

#### 2.2.2. Survey with Micro-XRF and SEM-EDS

**Figure 6:**
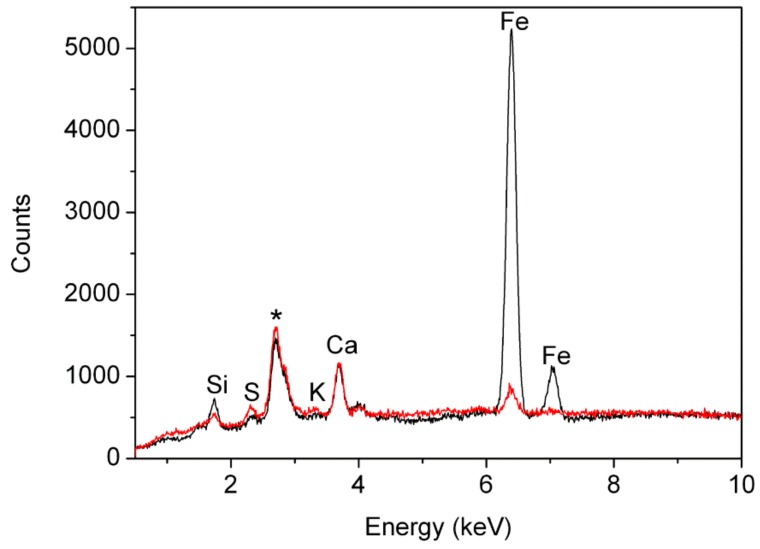
(Red) micro-XRF spectrum of sample BM2 selected as representative of samples dyed with organic dyes (madder or safflower); (black) micro-XRF spectrum of sample ME7, coloured with iron compounds. The peak marked with the asterisk arises from the Rh anode.

Micro-XRF analyses highlighted the presence of Si, S, Ca, and Fe in all the samples (Figure 6). Although no quantitative data were obtained, signal intensities for these elements were mostly comparable among the samples after normalisation to the intensity of the Rayleigh scattering radiation of the anode. Weak signals due to K were generally detected, except for samples ME2, BM5, and ME4. It was apparent that for this element the intensity of the signals varied significantly within the same textile, as different results were obtained for the pairs of samples taken from the same textile. Samples BM1 and ME7 stood out from this general picture, as the intensity of the Fe signals was significantly higher than the intensity detected in all the other samples (Figure 6), in agreement with red ochre being the main colourant present in these cases. 

EDS analyses enabled a more selective approach. Despite the presence of a large variety of particles, preliminary imaging performed through the OM of the fibres allowed the electron beam to be focused selectively on the coloured particles. In addition, the areas of the fibres showing no evident contamination and some particles not connected to the colour were also considered during the analysis, for comparison.

**Figure 7:**
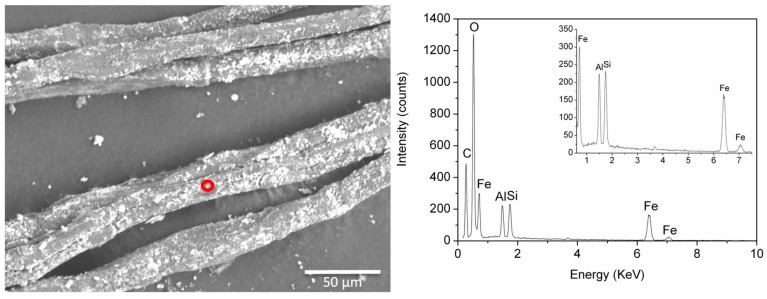
Back scattered electron SEM image of sample ME7 with an EDS spectrum obtained from the bright particles circled in red. Signals from the inorganic components are highlighted in the inset.

EDS analyses of samples BM1 and ME7 confirmed the presence of a fine dispersion of Fe-rich particles, which appeared bright in the back scattered electron images (Figure 7). In all the spectra recorded from these particles, Al, Si, and Ca were also detected, possibly pointing to a siliceous component. These elements were also detected in other particles poorer in Fe, suggesting the use of very fine ochreous earths for colouring the textile.

**Figure 8:**
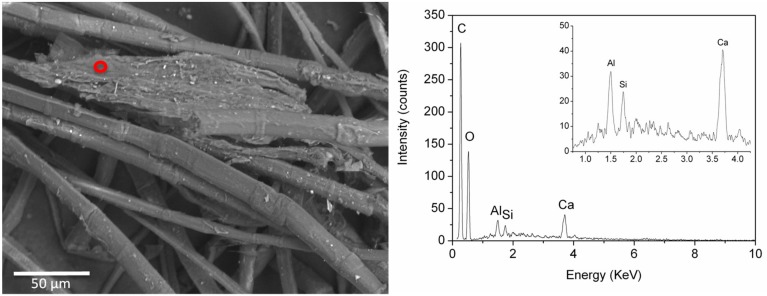
Back-scattered electron SEM image of sample BM4 with an EDS spectrum obtained from the coloured structures. Signals from the inorganic components are highlighted in the inset.

Regarding the samples dyed with madder, many spot analyses were performed on the madder-rich structures (Figure 8). Attention was paid to focus the beam only on the coloured aggregates, excluding the areas in which other particles were evident. Unfortunately, this was not possible for sample BM5, where the fibres were heavily contaminated. Therefore, elemental data for this sample will not be considered further. On the other hand, the analyses performed on the other three samples dyed with madder—ME2, BM2, BM4—produced useful qualitative information. The results were generally characterised by relatively low—and highly variable—peak intensities for all the detected elements, except for C and O, thus suggesting a mostly organic composition (Figure 8). Very reproducible spectra were obtained on areas of the fibres showing no evident contamination, where only intense peaks of C and O were detected (not shown). The analyses on the red agglomerates enabled Al, Si, and Ca to be detected in all the considered spots (Figure 8, inset) and P in most of them. In a few cases (not reported), S, Cl, and K were also detected in the EDS spectra.

Although the variability of the signals was certainly high, in samples BM4 and ME2, the signal from Al was always more intense than that from Si (Figure 8). It is suggested in the literature that, as Si is generally the most common contamination element, an Al/Si ratio >0.5 (in terms of peak intensity) can be taken as an indication of the presence of alum used as a mordant to fix the colour [31,46]. In the other samples, the Al/Si ratio was too variable to support any hypotheses and we could not exclude that the detected elements were mostly related to dust, clay, or other materials from the environment.

Although Fe signals were present in all the spectra recorded by micro-XRF, the possible role of this metal in the dyeing procedure was excluded by EDS, as Fe was not detectable in any of the red aggregates. On the other hand, both Ca and Al were always detected in the coloured structures. This may support the hypothesis of the formation of ternary complexes with the anthraquinones from madder, as suggested by Delamare et al. [47], in relation to alizarin fixed on cellulosic fibres, with the calcareous water of the dye bath as a possible source for the Ca ions. 

**Figure 9:**
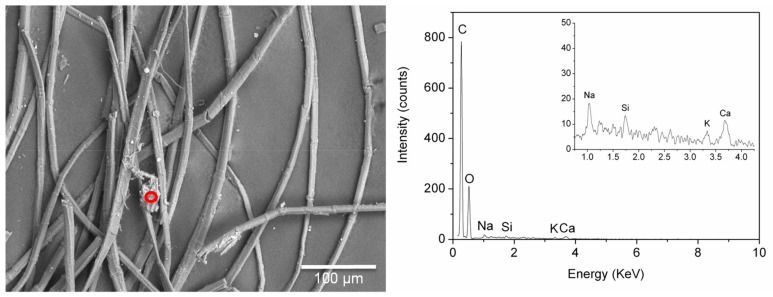
Back-scattered electron SEM image of sample BM3 and EDS spectrum obtain by focussing the electron beam on a coloured particle circled in red. Signals from the inorganic components are highlighted in the inset.

The definition of a reliable elemental composition for the coloured particles associated with safflower was even more difficult. In the cases in which it was possible to focus the beam on (apparently uncontaminated) coloured particles, they proved to be constituted mainly by C and O, although Ca and Na signals were detected in some analytical spots (Figure 9). The analytical data are in agreement with a direct dye, such as red safflower, being used without the addition of a mordant.

## 3. Discussion

The colourants identified in this study are consistent with those expected in textiles from the Pharaonic period. Colouring linen with natural earths rich in hematite has a long tradition in ancient Egypt [4,48]. Madder is indicated as the most important red dye of plant origin in the Dynastic period, during which safflower was also used as a source of red dye [1,4,48].

Nevertheless, the results obtained, especially in terms of morphological features and elemental composition, also enabled some hypotheses on the dyeing technologies and some considerations on the expected lightfastness of the colours to be formulated.

For the textiles coloured with red ochre, both the appearance of the textiles and the fine dispersion of the particles, which are evident on the fibres observed under the microscope, would exclude surface application in a way similar to painting or rubbing and would suggest, instead, some kind of soaking of the textile in a water-dispersion of red ochre, possibly with the aid of a medium. For these textiles, a very high durability of the colour is expected, as these inorganic red compounds are normally not prone to deterioration. In fact, the investigated red textiles coloured using red ochre exhibited a bright red colour.

On the other hand, most natural dyes are poorly to moderately colourfast [49] and the morphological properties of the dye, even more than the chemical structure, are among the most important intrinsic factors contributing to fading [50]. In the microscopic observations performed on samples dyed with madder, the coloured agglomerates embedding the fibres in some areas appeared to indicate that the colour derives from a process similar to the formation of a lake pigment, which is then deposited onto the textile fibres, rather than by chemical interaction between the dye molecules and the fibre surface, possibly mediated by metal ions or tannins. Fibres can be mordanted at different stages during the dyeing process [42]. Pre-mordanting, i.e. mordanting fibres prior to adding them to the dye bath, is the most common method, but post-mordanting and even the mixing of the mordant, fibres, and dye in the same dye bath are options. OM would suggest this latter method being used to dye the textiles under investigation. In fact, if madder is applied in a dye bath directly containing the mordant, the possibility of interaction between the mordant and the dye to create a lake, which then deposits on the fibres, cannot be excluded. In a few cases, S, Cl, and K were also detected in the EDS spectra, resembling those reported in the literature for madder lake pigments obtained by precipitating the dyes with potash alum [51,52]. Normally, fibres with large agglomerates of the dye are more light-fast, since a smaller surface area of the coloured material is exposed to air and light [53]. Nevertheless, the presence of coloured agglomerates that are not chemically bound to the fibres via a mordant results in these textiles being potentially prone to colour loss as a consequence of mechanical action. These two effects may explain why the red colour of the investigated textiles dyed with madder is relatively well-preserved in some cases and only partially preserved in other cases.

Safflower is a direct dye, whose fugitive nature is well-known [23,44,49]. In the samples under investigation, only a few small agglomerates of the dye were visible, and the dye appeared mainly adsorbed on the fibres. Carthamin adsorbed on textile fibres fades upon exposure to light in aerobic conditions because of the photo-oxidation of carbon-carbon double bonds [54]. This would explain the evident fading of the textiles dyed with safflower in those parts that have been exposed to light, whereas the colour has been—at least partially—preserved where the red textile has been protected from direct light.

## 4. Materials and Methods

Both FORS and p-FL spectra were recorded in situ by placing the probe holder directly over the mummy shrouds or onto the textile fragments, according to previously developed procedures [16,21,23]. On two mummies—namely, C. 2231/2 and C. 2233/1—both coloured and faded areas were considered.

Two FORS instruments were used, one at the British Museum and the other at the Museo Egizio. Both instruments were Avantes (Apeldoorn, The Netherlands) AvaSpec-ULS2048XL-USB2 spectrophotometers equipped with an AvaLight-HAL-S-IND tungsten halogen light source connected with a fibre optic bundle to an FCR-7UV200-2-1.5 × 100 probe. Light was sent and retrieved with the same fibre optic bundle, and the probe was set in a holder that kept it at a fixed distance of 2 mm (i.e., the focal distance of the probe) and with an angle of 45° with respect to the surface. This arrangement produced an analytical spot on the sample of 1 mm. In the case of the instrument from the Museo Egizio, a USB endoscope to visualise the investigated area was also fixed to the probe holder. As per the features of the monochromator (slit width 50 µm, grating of UA type with 300 lines/mm) and of the detector (2048 pixels), the best spectral resolution was 2.4 nm calculated as full width at half maximum (FWHM). Spectra were referenced against the WS-2 reference tile provided by Avantes guaranteed to be reflective at 98% in the considered spectral range. Although the spectral range of the detector extended from 200 to 1600 nm, the low emission of the illuminator effectively restricted the spectra to within 300 and 900 nm. The instrumental parameters were as follows: 10 ms integration time, 100 scans for a total acquisition time of 1.0 s for each spectrum. The whole system was managed by the AvaSoft v. 8TM software (Avantes) for Windows™. Spectra were acquired in reflectance mode (R) and then transformed into apparent absorption spectra by plotting the log (1/R).

p-FL was performed using an Ocean Optics Jaz model spectrophotometer equipped with a 365 nm Jaz-LED internal light source. The spectrophotometer responds in the range 191–886 nm with a spectral resolution of 7.6 nm, calculated as FWHM according to the features of the monochromator (groove density 600/mm, 200 µm slit width) and of the detector (2048 elements). A QF600-8-VIS/NIR fibre fluorescence probe was used to provide excitation light on the sample and to recover the emitted light. The probe was mounted on a holder that keeps it at a fixed distance of 12 mm (i.e., the focal distance of the probe) and with an angle of 45° with respect to the surface. This arrangement produced an analytical spot on the sample of 1 mm. A USB endoscope to visualise the investigated area was also fixed in the probe holder. The following instrumental parameters were employed: 4 s integration time, 3 scans. The system was managed by the SpectraSuite software (Ocean Optics).

OM was performed after placing the samples on a microscope glass slide without any pre-treatment. An Olympus BX51 transmitted and reflected light microscope—equipped with a halogen–quartz–tungsten lamp and an episcopic-fluorescent illuminator—was used to image the fibres under visible and UV light, respectively. The images were recorded by a digital camera Olympus DP71, performing both acquisition and processing by means of the Olympus AnalySIS software.

The samples subjected to SEM-EDS investigations were prepared by spreading the sample fibres over a carbon conductive double-sided adhesive tape fixed onto a SEM specimen stub. Prior to the SEM-EDS investigation, a Leica MZ 9.5 stereomicroscope was used to obtain preliminary colour images of the fibres attached to the stub in order to locate the coloured particles to be targeted with the analytical beam. A JSM IT300LV (JEOL USA) variable pressure (10/650 Pa) scanning electron microscope was used. The microscope was equipped with an INCA Energy 200 Energy Dispersive System mounting an INCA X-act SDD thin window detector and was managed by the INCA 21b SP3 software (all by Oxford Instruments). The preferred accelerating voltage 15 kV and a chamber pressure of 30 Pa were selected. The working distance was varied from 7 to 12 mm.

The micro-XRF equipment used was an Eagle III-XPL by Rontgenanalytik (Messtechnik GmbH, Germany), which combines an optical microscope to a micro-beam XRF analyser. The system includes a Rh X-ray tube working at a maximum voltage of 50 kV and a maximum current of 1 mA. The X-ray fluorescence is detected by means of a thermoelectrically cooled Si-drift detector with an active area of 30 mm^2^ and a 5 μm thick Beryllium window. Poly-capillary lenses collimate the X-ray micro-beam at the sample surface with a beam spot of about 30 μm. The analytical conditions were as follows: Voltage, 30 kV; beam current, 300 μA; live time, 100 s. All analyses were carried out under vacuum.

For HPLC-ESI-Q-ToF analyses, each sample was prepared by treating approximately 2–3 mm (ca. 100 µg) of a single thread using the method published elsewhere [16,40,53,55], which consists of a double mild extraction procedure using DMSO first and then a mixture of methanol/acetone/water/0.5 M oxalic acid 30:30:40:1 (v/v/v/v).

The solutions were analysed by a 1260 Infinity HPLC (Agilent Technologies), coupled to a Quadrupole-Time of Flight tandem mass spectrometer 6530 Infinity Q-ToF detector (Agilent Technologies) by a Jet Stream ESI interface (Agilent Technologies), as detailed in [16,39,46,55]. Briefly, a Zorbax Extend-C18 column was used with a 0.4 mL/min flow rate and 40°C column temperature. Separation was achieved using a gradient of water with 0.1% formic acid and acetonitrile with 0.1% formic acid. High-resolution MS and MS/MS spectra were acquired in negative mode in the range 100–1700 m/z. For the MS/MS experiments, different voltages (from 10 to 40 V) in the collision cell were tested for collision-induced dissociation (CID), in order to maximise the information obtained from the fragmentation.

Methanol (Sigma Aldrich, HPLC grade, purity ≥99.9%), acetonitrile (VWR, HiPerSolv CHROMANORM, HPLC grade, purity ≥99.9%), acetone (Fisher Scientific, pesticide residue grade, purity ≥99.8%), dimethylsulphoxide (AnalaR, purity ≥99.5%), oxalic acid dihydrate (AnalaR, purity ≥99.9%), and formic acid (Sigma Aldrich, eluent additive for LC-MS) were used as received.

The identification of the molecules in the samples was based on the comparison of the retention times and MS/MS spectra of standard molecules.

## 5. Conclusions

Both FORS and UV-OM microscopy proved to be very effective and affordable tools for an informative screening of the colouring materials. FORS can provide the conclusive identification of the main colouring agents, and UV-OM is able to distinguish between luminescing (madder and safflower) and absorbing (red ochre) materials, as well as provide preliminary information on the distribution of the colouring agents on the fibres and on the expected photo-stability of the red colour.

The information obtained by HPLC-MS enabled more details to be collected when organic dyes are present. The sensitivity of the technique is higher compared to the non-invasive approaches and mixtures of dyes can be easily identified.

The investigation of the inorganic components by comprehensive analysis on the whole fibre by micro-XRF and spot analysis on single particles by SEM-EDS enabled the presence of Fe to be confirmed in the case of red ochre and some indications of the presence of alum to be obtained. However, the unequivocal recognition of the raw materials possibly used for fixing the dyes proved to be a hard task, mainly because of general environmental contamination. Nevertheless, the combination of micro-morphological observations and elemental analyses yielded some insights into the colouring processes of these textiles, as described in the Discussion section. Considering what is reported in the literature, four dyeing techniques are mentioned as being used in Dynastic Egypt: “smearing” (with the colour literally spread onto the textile, possibly with the aid of a medium such as a clay, mud, or honey), vat dyeing (for indigo), mordant dyeing, and double-dyeing (to obtain mixed colours) [1]. Our results reveal a more complex picture and the need to focus future research not only on dye identification, but also on a better understanding of dyeing technology. 

This study therefore represents a window into dyeing technologies, availability of resources, and technical knowledge at a particular period in the history of Egypt and aids the elucidation of how these technical/material choices have contributed to colour or rather “uncolour” our view of the original intended appearance of these textiles. It is envisaged that such a robust dataset would be a good starting point from which to collect additional scientific data. It would also be of considerable value to archaeologists in assessing how the use of red shrouds and textiles was embedded in the funerary practices of ancient Egypt and how the colour of these textiles carried symbolic value that is now partially lost to us because of degradation processes. Finally, the information obtained in this study will be vital in aiding museum curators to plan the display of archaeological evidence and will further inform conservators not only about the risks of light exposure, but also about the risks of handling these fragile textiles, as mechanical abrasion can represent an additional threat.

## Figures and Tables

**Table 1:** Brief description of the textile fragments and mummies from the collections of the British Museum (BM) and the Museo Egizio (ME) under investigation in this study. Labels for the samples removed for investigation with micro-invasive techniques are also reported.

Reg. n.	Label	Description	Date (BCE)
EA 6523	BM1	Fragment of intense red linen cloth of medium quality.	18th dynasty–Ptolemaic period (ca. 1500–300)
EA 6518	BM2	Long strip of linen cloth with red straps taken from the mummy of a woman (Takush).	25th dynasty (ca. 700–650)
EA 6666	BM3	Mummy of a woman (name unknown). The outer shroud is bound by two vertical and four horizontal straps, with another strap passing round the neck and crossed over the breast. Underneath the straps an orange colour is noted.	Probably 26th dynasty (ca. 700–500)
EA 48971	BM4	Mummy of a woman (name unknown). The inner shroud appears red underneath the outer straps.	21st dynasty (ca. 1050–900)
EA 6669	BM5	Mummy of a man (Ameniryirt). The inner shroud appears red underneath the outer straps.	26th dynasty (ca. 600)
EA 6676	BM6	Mummy of a man (Penamunnebnesuttawy). The outer shroud is intact and is bound by two vertical and six horizontal straps, with another strap passing around the neck and crossed over the breast upon which is laid a semi-circular painted cartonnage pectoral. Underneath the straps an orange colour is noted.	25th dynasty (ca. 700)
C. 2215/2	ME1	Mummy of a woman (Tapeni). The outer shroud is intact and is bound by large straps crossed over the breast and passing around the body. The shroud appears red/pink underneath the straps and also the area of the body within the coffin.	25th dynasty (ca. 700–650)
S. 5227/2	ME2	Mummy of a man, “cultivator of lotus flowers of the Amun’s Temple” (Nesamonendjam). The outer bright red shroud is fragmented and only the lower part is preserved. It covers legs and feet. Brown areas on may suggest the presence of some straps (made of leather or metal) now disappeared, used to fix the shroud to the body.	25th dynasty (ca. 700–650)
C. 2231/2	ME3 and ME4	Mummy of a woman (Renpetnefret). The outer shroud is intact and is bound by large straps crossed over the breast and passing round the head and over knees. Other smaller straps are around the ankles and the feet. The shroud appears red/pink underneath the straps and also the area of the body within the coffin. Two samples from coloured (ME3) and faded (ME4) areas were taken.	25th dynasty (ca. 700–650)
C.2233/1	ME5 and ME6	Mummy of a priest (Padiemenemipet). The outer shroud is intact and is bound by straps crossed over the breast, passing around the head, over the knees, around the ankles and the feet. The shroud appears red underneath the straps and also in the area of the body within the coffin. Two samples from coloured (ME5) and faded (ME6) areas were taken. The mummy is covered with a bead-net.	25th dynasty (ca. 700–650)
S. 1698	ME7	Piece of a bright red cloth, probably a shroud, from the Deir el-Bahri cachette (DB 320). Mummy unknown.	18th–21st dynasty (ca. 1500–1000)

**Table 2:** Summary of the results obtained in this study *.

Reg. n. (Label)	Non-invasive		Micro-invasive					
	FORS	p-FL	VIS-OM	UV-OM	HPLC-ESI- Q-ToF	µ-XRF***	SEM-EDS	
EA 6523 (BM1)	Red ochre	n.p. **	Homogeneous red colour	Fine dispersion of UV-absorbing particles	No dyes detected	Si, P, S, K, Ca, **Fe**	Abundant Fe on coloured particles; Al, Si and Ca generally present	
EA 6518 (BM2)	Madder	n.p. **	Light red homogeneous colour	Weak orange luminescence of some fibres/ agglomerates	Madder, tannins	Si, S, K, **Ca**, **Fe**	Abundant C and O and variable peak intensities of Al, Si, Ca, S, K and Cl on agglomerates	
EA 6666 (BM3)	Safflower	n.p. **	Light pink homogeneous colour	Intense orange luminescence of a few areas	Safflower	Si, K, Ca, Fe	Mostly C and O with low intensity of Na, Si, Ca, S, P and Cl	
EA 48971 (BM4)	Madder	n.p. **	Orange/red heterogeneous colour	Intense orange luminescence of the coloured agglomerates	Madder, tannins	Si, P, S, K, **Ca**, **Fe**	Abundant C and O and variable peak intensities of Al, Si, Ca, S, K and Cl on coloured agglomerates	
EA 6669 (BM5)	n.p.*	n.p. **	Dark red heterogeneous colour	Intense orange luminescence of the coloured agglomerates	Madder, tannins	Si, S, **Ca**, Fe	High level of contamination	
EA 6676 (BM6)	n.p.*	n.p. **	Pink homogeneous colour and red particles	Low intensity orange luminescence of some fibres and areas	Safflower	Si, S, K, Ca	Mostly C and O with low intensity of Al, Si, Ca, S, Na, P, Mg and Cl	
C. 2215/2 (ME1)	Safflower	Safflower	Light orange/pink homogeneous colour	Low intensity orange luminescence of some fibres and areas	Safflower	Si, S, K, **Ca**, **Fe**		Mostly C and O with low intensity of Al, Si, Ca, S, Na, P, Mg and Cl
S. 5227/2 (ME2)	Madder	Madder	Bright red heterogeneous colour	Dark orange luminescence of coloured structures	Madder, tannins	Si, S, **Ca**, Fe		Abundant C and O and variable peak intensities of Al, Si, Ca, S, K and Cl on coloured agglomerates
C. 2231/2, coloured (ME3)	Safflower	Safflower	Very light orange homogeneous colour	Light orange luminescence of some areas	Safflower	Si, S, K, Ca, Fe		Mostly C and O with low intensity of Al, Si, Ca, S, Na,, Mg and Cl
C. 2231/2, faded (ME4)	Not conclusive	Not conclusive	Natural flax colour	Whitish luminescence from natural flax	No dyes detected	Si, S, **Ca**, Fe		Mostly C and O with low intensity of Al, Si, Ca, S, Na, P, Mg and Cl
C. 2233/1, coloured (ME5)	Safflower	Safflower	Pink and orange heterogeneous colour	Orange luminescence of some areas	Safflower	Si, S, K, Ca, Fe		Mostly C and O with low intensity of Ca
C. 2233/1, faded (ME6)	Not conclusive	Not conclusive	Natural flax colour	Very light orange luminescence of some fibres	Safflower (traces)	Si, S, K, Ca, Fe		Mostly C and O with low intensity of Ca and Na
S. 1698 (ME7)	Red ochre	Not conclusive	Homogeneous red colouration of the fibres	Fine dispersion of UV-absorbing particles	No dyes detected	Si, P, S, K, Ca, **Fe**		Abundant Fe on coloured particles; Al, Si and Ca generally present

* As part of a separate study, the shroud of the Nestawedjat mummy (25th dynasty, EA 22812, British Museum) was also analysed by HPLC-MS and found to be dyed with safflower [35]. ** not performed; *** elements related to the most intense peaks are reported in bold.
